# Perspective on the role of norms for institutional behavior and policy design in European cross-border regions

**DOI:** 10.3389/fsoc.2024.1434306

**Published:** 2025-01-14

**Authors:** Sara Svensson

**Affiliations:** School of Education, Humanities and Social Sciences, Halmstad University, Halmstad, Sweden

**Keywords:** cross-border cooperation, border regions, regionalization, norms, values, multi-level governance

## Abstract

The article analyses how a norm scientific perspective can advance our understanding of cross-border regions and guide future directions of research. Cross-border regions are territorial spaces comprising territory from two or more national states, located directly at the borders of those spaces. Since the 1950s it has become increasingly common that cross-border organizations, constituted by local municipalities and regional authorities and sometimes private entities, are established to coordinate governance processes around shared policy problems. These organizations fit into a Type II model of European multi-level governance as complex, fluid, and carried out in overlapping jurisdictions. A norm scientific perspective focuses on joint expectations as a primary predictor of behavior and thereby on social structures as well as social transformations. In accordance with institutional theory, norms are understood as intersubjective, widely shared, but often implicit, expectations and rules that guide human behavior. The article makes two arguments. First, it argues that a norm-scientific perspective has the potential to significantly advance the scientific community's understanding of various aspects related to how cross-border cooperation emergence and functioning. Second, it argues that cross-border regions constitute a promising venue to advance the knowledge of how norms can be studied and understood.

## Introduction

Humanity has an enduring tendency to set, maintain and defend borders. Typical of the Westphalian international order, developed from the 17th century onwards, is the focus on precise delimitations of national borders. This has created all sorts of situations for people living in the borderlands. In Europe, up to a third of European Union (EU) inhabitants live in what the EU officially classified as borderlands: NUTS 3 regions located next to the border with another nation, including entire nation-states such as Luxembourg.^1^ However, in the context of power shifting away from national arenas to new territorial and non-territorial spaces, national borders have increasingly been seen as non-optimal or arbitrary, and there has been a remarkable shift toward addressing policy issues derived from that inadequacy with non-conflictual means. One manifestation on the ground is the proliferation of local cross-border alliances of public authorities, something which is especially notable in Europe (Brunet-Jailly, 2022).

In line with the scope of Perspectives Articles, the purpose of this article to present my viewpoint on a specific area of investigation, in this case my take on how *a norm scientific perspective* can advance our understanding of crossborder regions and crossborder regional organizations and guide future directions of research.

A norm scientific perspective (e.g., Hydén, 2022; Svensson M., 2013) focuses on joint expectations as a primary predictor of behavior and thereby on social structures as well as social transformations. In accordance with institutional theory (Scott, 1987, 2008), norms are understood as intersubjective, widely shared, but often implicit, expectations and rules that guide human behavior (Svensson M., 2013). The article makes two arguments. First, it argues that a norm-scientific perspective has the potential to significantly advance the scientific community's understanding of various aspects related to how cross-border cooperation emerge and function. Second, it argues that cross-border regions constitute a promising venue to advance the knowledge of how norms can be studied and understood.

## Norms and the creation of cross-border institutions in European borderlands

Cross-border regions are territorial spaces comprising territory from two or more national states, located directly at the borders of those spaces. It has become increasingly common in many parts of the world that cross-border organizations, constituted by local municipalities and regional authorities and sometimes private entities, are established to coordinate governance processes around shared policy problems. This is especially the case in Europe, where this development started in the 1950s and where these are referred to by varying terms and take different legal forms, but are often colloquially referred to as Euroregions. In 2006, the introduction of a joint European judicial format, the European Groups of Territorial Cooperation, EGTCs (Svensson and Ocskay, 2015; Engl, 2016), constituted an important step in this regard.

**Figure 1:**
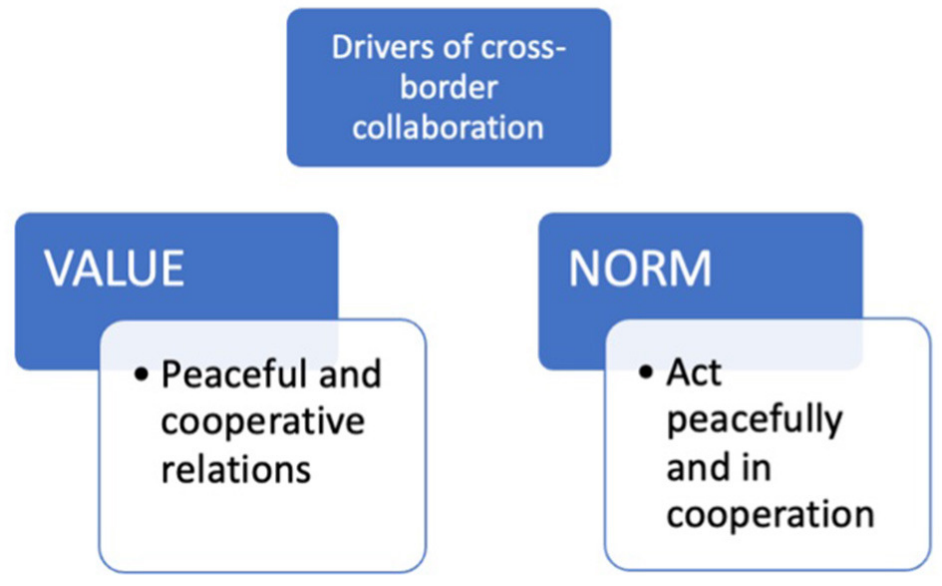
Drivers of European cross-border collaboration.

Generally, cross-border cooperation organizations fit into a Type II model of European multi-level governance as complex, fluid, and carried out in overlapping jurisdictions (Marks and Hooghe, 2004). They were often founded based on the expectation that such organizations can contribute to peace and prosperity; the assumption is that cooperation facilitates cross-border mobility of goods, services, and people, which in turn is expected to lead to economic growth and social cohesion. Later, peaceful and cooperative relations began to be seen as a European value in itself, i.e. to act peacefully, and in cooperation, became a European norm (see Figure 1).

Research by the author has previously demonstrated the prevalence of this type of value-based driver for the emergence of cross-border cooperation organizations at borderlands characterized by cultural and linguistic similarity, whereas the more instrumental end-goal of economic development has been more important in cross-border territories characterized by asymmetry, such as those located along the former cleavage between Western and Eastern Europe. As an example, Euroregions at the Austrian-German border display EU prominently in virtually all communication and policy discourses and representatives of border committees at the Norwegian-Swedish border see cooperation as something of intrinsic value seen in a Scandinavian context. On the other hand, grant access motivations featured prominently at the German-Polish border (Medve-Bálint and Svensson, 2012; Medve-Bálint, 2013; Svensson S., 2013).

The increase in the number of Euroregions also follows a trajectory of policy transfer from North/West to South/East Europe, pushed for by a set of actors such as the European Union, the Council of Europe, the regional Nordic Council, and in the case of Central East Europe, the inter-government collaboration Visegrad Four and the think tank CESCI. They were key to the transmission of a basic format (the Euroregion as an organization), underpinned by the above-mentioned values expressed as policy goals (peace and prosperity), and norms (collaboration across borders and nations as an important mechanism to reach those goals). However, as I have argued elsewhere (Svensson, 2018, p. 141), the original norms were reinterpreted through a process of normative and mimetic institutional isomorphism (DiMaggio and Powell, 1983). The idea of “peace through cooperation,” implying an inclusive approach, became something often used for kinship-based cooperation, such as between municipalities with Hungarian-speaking populations in countries bordering Hungary: “the Euroregion, as a transferred policy institution, was not only a vehicle for EU aspirations but also ‘borrowed' (or hijacked) and adapted for an additional, or even contrary, set of policy objectives” (Svensson, 2018, p. 141).

Thus, a norm scientific perspective can help us understand why cross-border cooperation in the form of Euroregional organization-formation became so common in Europe in the second half of the 20th century. The motivational drivers behind Euroregions are often shared values and norms, or the aspiration of certain values and norms. Once they are formed, however, a question of interest is how norms matter with relation to policymaking, which is the focus of the next section.

## Norms and policymaking in European borderlands

“We cannot do that because of the rules.” This sentence, or variations thereof, is often said by actors representing Euroregions or actors in other ways involved in cross-border cooperation. It can be uttered by a local mayor representing a municipal member of a Euroregion talking about the benefits that could be gained by coordinating elderly care in peripheral border settlements, or stated by a Euroregional project manager explaining why school children cannot (easily) attend a school on the other side of the border. What these actors mean when they talk about “rules” is not only a broad spectrum of formal norms (laws and regulations) that differ between the two or more nation states to which the borderland territory belongs. It also refers to the entire complex institutional compound of hierarchical and vertical competence distribution across multi-level governance systems, and the asymmetry effects caused by that. Euroregions rarely have decision-making powers in their own right; to have effect on policy and everyday life in the borderlands they are dependent on local, regional, national and EU authorities.

This explains to some extent differences in assessment when it comes to what Euroregions have achieved, beyond spreading as a type of organization and bringing people together. However, in general, researchers tend to be cautious when assessing their overall capacity (Perkmann, 2003; Svensson S., 2013; Opilowska et al., 2017; Telle and Svensson, 2020; Noferini et al., 2020). In recent years there has also been setbacks due to a general shift toward framing borders in security terms, as protection from unwanted migration, crime, or viruses. Perhaps more interesting, however, is to take a holistic approach to cross-border policy-making and see what the effect of these multiple-actor-networks have been.

For this, a norm scientific perspective, based on institutional theory, is helpful, and has not so far been sufficiently used to understand performance within cross-border cooperation.

Norms are at the heart of the proliferation of a working method based on systematic identification of “obstacles” (sometimes also “drivers”). An early adopter of this way of working was the Nordic Council (comprising Denmark, Norway, Sweden, Finland and Iceland, as well as the autonomous territories of Faroe Islands, Greenland and Aland). The Nordic Council supported a methodology, whereby committees in borderlands started to identify and name factors that hindered flows across the border, i.e. obstacles. Often these would be in the form of differences in legal and regulatory systems, but also other issues—including cultural stereotyping—could be included. Originally, this was done within different regions, but since 2014 there is a Border Obstacle Committee, consisting of 10 members (8 national representatives, the general secretary of the Nordic Council of Ministers and a representative of the Nordic Council). The committee should collaborate closely with other stakeholders, such as private sector representatives, authorities, political bodies, and others. All obstacles are collected in a database (Nordic Council, 2024), where one can see the category and status, and whether it is prioritized, such as in the following example.

**Reimbursement for patient travel between Norway and Finland/Sweden**

A cross-border commuter residing in Finland or Sweden and working in Norway is socially insured and liable to tax in Norway. He or she does not receive compensation for their patient travel/treatment travel from home in Finland/Sweden to the hospital in Norway, unlike their work colleagues who live and work in Norway.

Affected/affected countries: Finland Norway Sweden

Category: Social and health

Status: Solved

Prioritized by the Border Barriers Council: No

Through a norm-scientific perspective, we see how these short statements, expressed in dry but accessible public administration prose, serves to (a) verbalize and make visible formal rules in order to show that they can be changed, and (b) point to action drawing on underlying values and norms related to the potential, possibility and promises of cross-border regional integration.

This approach or working methodology has subsequently been adopted by continental European actors at well (Medeiros, 2018). It was reinforced through an initiative launched in 2015 by the European Commission's Directorate General for Regional and Urban Policy (DG REGIO), aiming to identify border obstacles. Through a concerted effort by participants, representing European Union Member states and their regional and local authorities as well as other stakeholders, 239 obstacles were identified. Most of these concerned the labor market & education, social security and health or transport and mobility. The inventory was followed by a public consultation and a work, resulting in a publication disseminated broadly to stakeholders (European Commission, 2017). This has then been followed up by *b-Solutions*, an initiative that has continued this work, focusing on administrative and legal obstacles, and managed by the Association of European Border Regions. The obstacles are presented in a similar manner to that of the Nordic Council and covers issues of a broad range, but with a focus on legal obstacles.

**Boosting Minho River cross-border sharing services**

Advised entity: River Minho EGTC

In the Rio Minho border region, the first truly joint cross-border e-bike system between Spain and Portugal was established in 2022, reflecting the strong sentiment for cross-border cooperation in this densely populated region. Although the e-bike system is managed by the Rio Minho EGTC, which is a European legal entity with its own legal personality, tax regulations complicate the shared management of the project. In particular, it was necessary for the EGTC, which has its seat in Portugal, to set up a secondary office in Spain in order to comply with VAT requirements. Finding a solution to fiscal obstacles would greatly enhance the efficiency of such shared public services.

**Cross Border Internships**

Advised entity: Region Sønderjylland-Schleswig, Regionskotor and Infocenter.

Due to different legislative frameworks in Denmark and Germany, students and workers are limited in their opportunities to pursue internships on the other side of the border. It is problematic as cross-border internships can often lead to hiring and can thus strengthen the border region labor market.

In evaluations carried out by AEBR together with researchers, the B-solution has been largely positively evaluated (Medeiros et al., 2022, 2023). Notably, the method has been elaborated to include the allocation of an external expert to certain identified obstacles. This expert investigates the obstacle in detail and proposes solutions. These proposed solutions are then shared with the broader community via a web-based database. A further development of the method is that the origin of the obstacle is clearly identified and can serve as a focal point for further action.

The obstacle-based method has also been worked into a new important legal development, namely to solve the member state resistance to what would have been an important legal development, the 2018 EC proposal to establish a European Cross-border Mechanism (Engl and Evrard, 2020). The latter would entail the implementation of a daring anti-Westphalian, practice, namely for the application of legislation of one Member State in another Member State. Under certain conditions and with the agreement of the States in question, borderland citizens could in some respect therefore be subject of the same legislation, regardless of in which country they reside (Jankova et al., 2023; Rosanò, 2021). Since this was seen to threaten the sovereignty of member states over their territories, a reworked model called the Cross-Border Facilitation Tool will let Cross-border Coordination Points make assessments based on the obstacle-model and negotiate with member states in each case.

What we see is therefore how a conscious norm-based approach seeks to contribute to actual change in borderlands, through turning informal and formal norms that affect the borderlands from invisible to visible.

## Discussion and concluding remarks

This article outlined how the transformation of borderland regions into cohesive spaces is dependent on norms that carry them. Prevailing norms (informal and formal rules) can hinder this transformation and therefore serve as obstacles. New policy practice strategies in Europe aims at making these obstacles visible, and thereby contribute to the emergence of new norms, informal and formal, which would be conducive for the development of these new territorial spaces. Taken together, the article demonstrates how a norm-scientific perspective has the potential to significantly advance the scientific community's understanding of various aspects related to how cross-border cooperation organizations function, and also to impact development on the ground. It has also demonstrated that cross-border regions constitute a promising venue to advance the knowledge of how norms can be studied and understood.

Not dealt with in this article, but a prospect for further research with a norm scientific perspective is the question whether there are widely shared implicit rules and expectations in borderlands, which would yield a certain sense of predictability around acceptable standards of behavior. More knowledge around that would be especially pertinent in light of geopolitical events and developments that in recent years have challenged cross-border cooperation, including but not limited to the migration crisis of 2015, the COVID-19 pandemic and the Russian full-scale invasion of Ukraine and subsequent ongoing war at Europe's edges.

## Data Availability

The original contributions presented in the study are included in the article/supplementary material, further inquiries can be directed to the corresponding author.

## Footnotes

^1^This does not mean that all these inhabitants self-identify as *borderlanders*. In everyday-life, people tend to understand borderlands as more narrowly delimited spaces, such as tertiary-level administrative spaces located at the border or even spaces from which the border can be visibly seen.
